# Risk factors and a nomogram for bovine jugular vein conduit failure after right ventricular outflow tract reconstruction: a 10-year single-center cohort

**DOI:** 10.3389/fped.2026.1733522

**Published:** 2026-02-06

**Authors:** Junquan Chen, Jisheng Zhong, Junying Guo, Yunpeng Bai, Junwu Su, Zhigang Guo

**Affiliations:** 1 Department of Cardiovascular Surgery, Chest Hospital, Tianjin University, Tianjin, China; 2 Tianjin Medical University, Tianjin, China; 3 Pediatric Cardiology Center, Capital Medical University Affiliated Anzhen Hospital, Beijing, China; 4 TEDA International Cardiovascular Hospital, Tianjin University, Tianjin, China

**Keywords:** bovine jugular vein conduit, congenital heart disease, nomogram, prediction model, right ventricular outflow tract reconstruction

## Abstract

**Background:**

Bovine jugular vein conduit (BJVC) is widely used for right ventricular outflow tract (RVOT) reconstruction, yet long-term durability varies and individualized risk tools remain limited.

**Methods:**

We conducted a single-center retrospective cohort of consecutive children undergoing primary BJVC implantation between 2011 and 2020. The primary endpoint was BJVC failure, defined as surgical or catheter-based reintervention for conduit dysfunction or infective endocarditis. Freedom from failure was summarized at 1, 3, 5, and 7 years. Candidate predictors comprised demographics, pre-operative echocardiography/laboratory data, operative metrics (conduit internal diameter, cardiopulmonary bypass and cross-clamp times), and pre-discharge residual RVOT gradient. Missing data (0%–11%) were handled using multiple imputation by chained equations. Predictors selected by LASSO were entered into multivariable Cox regression. Model performance was evaluated for discrimination (Harrell's C; time-dependent AUC) and calibration, with internal validity assessed by bootstrap optimism correction. A nomogram was constructed to provide individualized 1-, 3-, 5-, and 7-year estimates of freedom from failure.

**Results:**

Seventy-eight patients were included (median follow-up, 7.7 years); 29 conduit failures occurred (37.2%). All conduit reinterventions were surgical redo procedures; no patient underwent catheter-based balloon dilation of the BJVC prior to surgical reintervention. Independent risk factors were pre-discharge residual gradient ≥20 mmHg (HR 18.67; 95% CI 7.43–46.94), longer cardiopulmonary bypass time (per 10-minute increase HR 1.28; 95% CI 1.15–1.43), male sex (HR 2.25; 95% CI 1.11–4.58), age ≤1 year (HR 1.76; 95% CI 1.09–2.86), and conduit diameter ≤14 mm (HR 1.68; 95% CI 1.04–2.74). The model demonstrated good discrimination (Harrell's C-index 0.82) and acceptable calibration; bootstrap internal validation yielded similar performance, with time-dependent AUCs of 0.888, 0.850, 0.900 and 0.897 at 1, 3, 5, and 7 years, respectively. Freedom from failure at 1, 3, 5, and 7 years was 97.4%, 87.2%, 75.6%, and 62.8%, respectively.

**Conclusions:**

Using routinely available peri-operative variables, we developed an interpretable nomogram to estimate the risk of BJVC failure and to inform individualized surveillance and intervention planning. The model showed good internal performance but was derived in a single-center cohort with a modest number of events; prospective multicenter validation and, if necessary, recalibration are required before routine clinical implementation.

## Graphical Abstract

**Figure:**
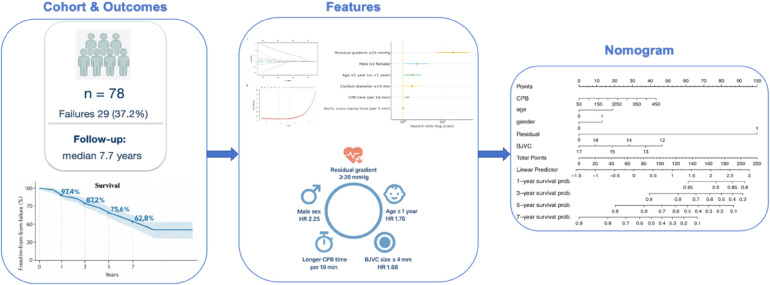

## Introduction

Right ventricular outflow tract (RVOT) reconstruction is fundamental to the surgical management of complex congenital heart disease (CHD) (1, 2). Among available substitutes, the bovine jugular vein conduit (BJVC) is widely adopted in infants and young children owing to its small-diameter availability, anatomical conformity (native trileaflet valve), and reproducible handling (3, 4). Nonetheless, long-term durability remains heterogeneous: progressive stenosis, regurgitation, somatic outgrowth, and conduit-related infective endocarditis drive reintervention and long-term morbidity, with real-world freedom from failure declining over time (4–8). This heterogeneity at the patient, procedure and center levels makes it difficult to plan surveillance and to determine the optimal threshold for catheter-based or surgical reintervention in routine care (9, 10).

Prior studies suggest that durability after right ventricular outflow tract reconstruction is influenced by factors present at or before discharge. Younger implantation age and smaller conduit diameter have been linked to earlier structural failure, prolonged cardiopulmonary bypass time reflects greater operative complexity, and higher early residual right ventricular outflow tract gradients signal unfavorable hemodynamics (11–14). These signals are biologically plausible because growth, conduit geometry, tissue–prosthesis interaction and early flow dynamics jointly shape subsequent stenosis, regurgitation and the need for reintervention. Yet most reports describe group-level associations rather than providing transparent, time-specific estimates that clinicians can apply to an individual child. In particular, there is a lack of simple, interpretable tools that combine routine peri-operative information to stratify risk and guide follow-up after bovine jugular vein conduit implantation.

Against this background, we sought to close the translational gap between group-level associations and bedside decisions by assembling a single-center cohort of children who underwent primary bovine jugular vein conduit implantation. Our aims were to identify independent predictors of conduit failure and to build an interpretable risk tool that estimates freedom from failure at clinically relevant time points, using only information available by discharge and evaluating performance through discrimination and calibration with a conservative bootstrap-based internal validation strategy.

## Methods

### Study design and setting

We conducted a retrospective, single-center cohort study at a tertiary congenital heart center. Consecutive pediatric patients undergoing primary bovine jugular vein conduit (BJVC) implantation for right ventricular outflow tract (RVOT) reconstruction between January 2011 and December 2020 were eligible. The protocol (IRB Approval No. 2025031X) complied with the Declaration of Helsinki. Clinical care and data abstraction followed center-wide standard operating procedures current to the study period.

### Participants

#### Inclusion criteria

(1) Age ≤18 years; (2) first-time BJVC implantation for RVOT reconstruction; (3) available follow-up ≥6 months or until a qualifying endpoint.

#### Exclusion criteria

(1) Peri-operative mortality within 30 days; (2) prior RVOT conduit or valve prosthesis; (3) missing primary-outcome data or loss to follow-up; (4) concomitant implantation of a non-BJVC valve in the RVOT at index surgery.

Indications encompassed complex congenital heart disease requiring RVOT reconstruction (tetralogy of Fallot, pulmonary atresia, truncus arteriosus, double-outlet right ventricle, and transposition of the great arteries with VSD/PS).

### Operative technique and peri-operative management

Operations were performed via median sternotomy under standard cardiopulmonary bypass (CPB) with moderate hypothermia (28–32°C). Myocardial protection used histidine–tryptophan–ketoglutarate (HTK) cardioplegia after aortic cross-clamping. Glutaraldehyde-fixed BJVCs were rinsed in sterile saline; diameter selection targeted 2–4 mm above the expected native pulmonary-artery diameter to accommodate growth. Conduits were trimmed to avoid kinking, with a bevelled distal end to enlarge the pulmonary-artery anastomosis. Anastomoses were fashioned to avoid purse-stringing; the conduit was positioned proximally near the pulmonary bifurcation and protected from compression by the ascending aorta and sternum at closure. Post-operatively, patients received intravenous heparin (5–15 U/kg/h) transitioned to aspirin 5 mg/kg once daily for 1 year unless contraindicated.

### Follow-up and echocardiography

Outpatient follow-up followed institutional practice. Transthoracic echocardiography was performed by experienced pediatric sonographers using standardized pediatric protocols; Doppler-derived peak velocity/gradient and related indices were averaged over ≥3 cardiac cycles in calm or sedated conditions when needed.

### Outcome

The primary endpoint was BJVC failure defined as reintervention (surgical or catheter-based) for conduit dysfunction or conduit-related infective endocarditis adjudicated by a multidisciplinary team (new vegetation on the conduit and/or positive blood cultures for typical organisms). Time-to-event was measured from implantation to first failure; patients without events were censored at last contact. Deaths clearly unrelated to the conduit were censored at the date of death.

A pre-specified sensitivity endpoint additionally considered “severe hemodynamic dysfunction at institutional echocardiographic thresholds”—Doppler instantaneous peak gradient >60 mmHg and/or regurgitation ≥3+—to examine robustness of findings.

### Candidate predictors

To preserve bedside applicability, pre-specified predictors available at or before discharge were: age, sex, weight/body-surface area, primary diagnosis and prior palliation, BJVC internal diameter (mm), CPB time (min), aortic cross-clamp time (min), and pre-discharge residual RVOT gradient on echocardiography, which was recorded in two categories (<20 vs. ≥20 mmHg) according to the institutional echocardiographic reporting system. Post-discharge or time-varying covariates were not used for model development. Continuous predictors were modeled on their original scales; where appropriate, restricted cubic splines were used to allow for nonlinearity.

### Missing data

Variables with >30% missingness were excluded *a priori*. Remaining variables (overall missingness ∼0%–11%) were imputed under a missing-at-random assumption using multiple imputation by chained equations (MICE) (15): predictive mean matching for continuous variables and appropriate multinomial models for categorical variables; unordered factors used one-hot encoding. Passive imputation was applied for derived terms. Estimates across imputed datasets were combined using Rubin's rules (16). The distributions of missingness and post-imputation diagnostics are shown in Supplementary Figure S1.

### Statistical analysis

#### Descriptive statistics

Categorical variables were summarized as counts (%); continuous variables as mean ± SD or median (IQR), as appropriate. Between-group comparisons used *χ*^2^ or Fisher's exact tests for categorical data and *t* tests or Mann–Whitney/Kruskal–Wallis tests for continuous data.

#### Prediction model development (single prespecified pathway)

To avoid information loss from dichotomization and reduce overfitting, we adopted a single prespecified modeling pathway. All candidate predictors were entered simultaneously after standardization. LASSO (glmnet, R) was used as an embedded feature-selection/regularization procedure (17). The penalty parameter λ was selected by cross-validation, with coefficient-path visualization across log (λ). Predictors with non-zero coefficients at the selected *λ* constituted the final set for modeling. We then fitted a Cox proportional hazards model to estimate adjusted hazard ratios (HRs) with 95% confidence intervals for interpretability (18). For time-scale clarity, effects for duration-type variables were additionally reported per 10 min increase. Proportional-hazards assumptions were assessed using Schoenfeld residuals and visual diagnostics; if violated, time-varying effects [interaction with log(time)] or stratification were considered. Given that 29 conduit failures occurred and five predictors were retained in the final multivariable model, the resulting events per variable (EPV) was approximately 5.8. This is below the commonly recommended threshold of 10–15 EPV for prediction model development. We therefore relied on penalized variable selection (LASSO) and bootstrap internal validation to mitigate overfitting and interpreted the model as exploratory rather than definitive.

#### Internal validation and model performance

Internal validity was assessed using bootstrap optimism correction (≥1,000 resamples) applied to the full model-building pipeline, including variable selection. Discrimination was quantified by Harrell's C-index and ROC-based measures. A conventional ROC curve was used to obtain the apparent AUC for predicting freedom from conduit failure over the entire follow-up, and time-dependent ROC analysis was performed to estimate AUCs at 1, 3, 5, and 7 years, using the linear predictor of the multivariable Cox model as the risk score (timeROC package in R). Calibration was evaluated by calibration-in-the-large and calibration slope, together with flexible time-specific calibration plots at predefined time points. A nomogram (rms package) was constructed to estimate individualized 1-, 3-, 5-, and 7-year freedom from failure probabilities. These procedures were intended to reduce optimism in performance estimates in the context of the relatively low events-per-variable ratio.

#### Sensitivity analyses

We repeated model fitting using the sensitivity endpoint (adding severe echocardiographic dysfunction) and compared effect sizes, discrimination, and calibration with the primary endpoint. Additional sensitivity analyses explored alternative functional forms and complete-case analyses.

#### Software and thresholds

Analyses were performed with SPSS 26.0 and R 4.4.2 (key packages: glmnet, survival, rms, mice, timeROC, rmda). Two-sided *P* < 0.05 was considered statistically significant; where applicable, 95% confidence intervals were reported.

## Results

### Cohort characteristics

From January 2011 to December 2020, 87 children underwent primary BJVC implantation. After excluding peri-operative deaths (*n* = 4, 4.6%) and loss to follow-up (*n* = 5, 5.7%), 78 patients were analyzed. All conduit reinterventions were surgical redo procedures; no patient underwent catheter-based balloon dilation of the BJVC prior to surgical reintervention. Median follow-up was 7.7 years; no late deaths occurred. The cohort showed a slight male predominance (44/78, 56.4%) and a young age profile (median age 20 months; range 7–156), with 30/78 (38.4%) aged ≤1 year; 41/78 (52.6%) had prior palliation. Primary diagnoses were pulmonary atresia (55/78, 70.5%), TGA/VSD/PS (9/78, 11.5%), DORV (6/78, 7.7%), truncus arteriosus (5/78, 6.4%), and TOF (3/78, 3.8%). Conduits >14 mm were used in most cases (62/78, 79.5%; ≤14 mm in 16/78, 20.5%). Mean CPB and aortic cross-clamp times were 147.3 ± 36.2 and 75.2 ± 26.4 min, respectively. Early postoperative course included 49.6 ± 20.4 h of mechanical ventilation and 118.4 ± 56.7 h in the ICU. Early complications were infrequent: delayed sternal closure 3/78 (3.9%), re-exploration 4/78 (5.2%), and complete atrioventricular block 2/78 (2.6%). Full baseline and operative details appear in Table 1.

**Table 1:** Cohort profile, baseline characteristics, operative details (*n* = 78).

Characteristic/variable	Value
Baseline characteristics	
Male (%)	44 (56.4)
Age at implantation (month)	20 (7–156)
Age ≤1 year (%)	30 (38.4)
Primary pre-operative diagnosis (%)	
PA	55 (70.5)
TGA/VSD/PS	9 (11.5)
DORV	6 (7.7)
Truncus arteriosus	5 (6.4)
TOF	3 (3.8)
Pre-operative oxygen saturation (%)	83 (71–99)
Operative details	
Conduit diameter group (%)	
≤14 mm	16 (20.5)
>14 mm	62 (79.5)
CPB time (min)	147.3 ± 36.2
Aortic cross-clamp time (min)	75.2 ± 26.4
Mechanical ventilation (h)	49.6 ± 20.4
ICU stay (h)	118.4 ± 56.7
Early complications, *n* (%):	
Delayed sternal closure	3 (3.9)
Re-exploration	4 (5.2)
Complete atrioventricular block	2 (2.6)

BJVC, bovine jugular vein conduit; PA, pulmonary atresia; TGA, transposition of the great arteries; VSD, ventricular septal defect; PS, pulmonary stenosis; DORV, double-outlet right ventricle; TOF, tetralogy of Fallot; ICU, intensive care unit; CPB, cardiopulmonary bypass.

### Survival analysis

**Figure 1:**
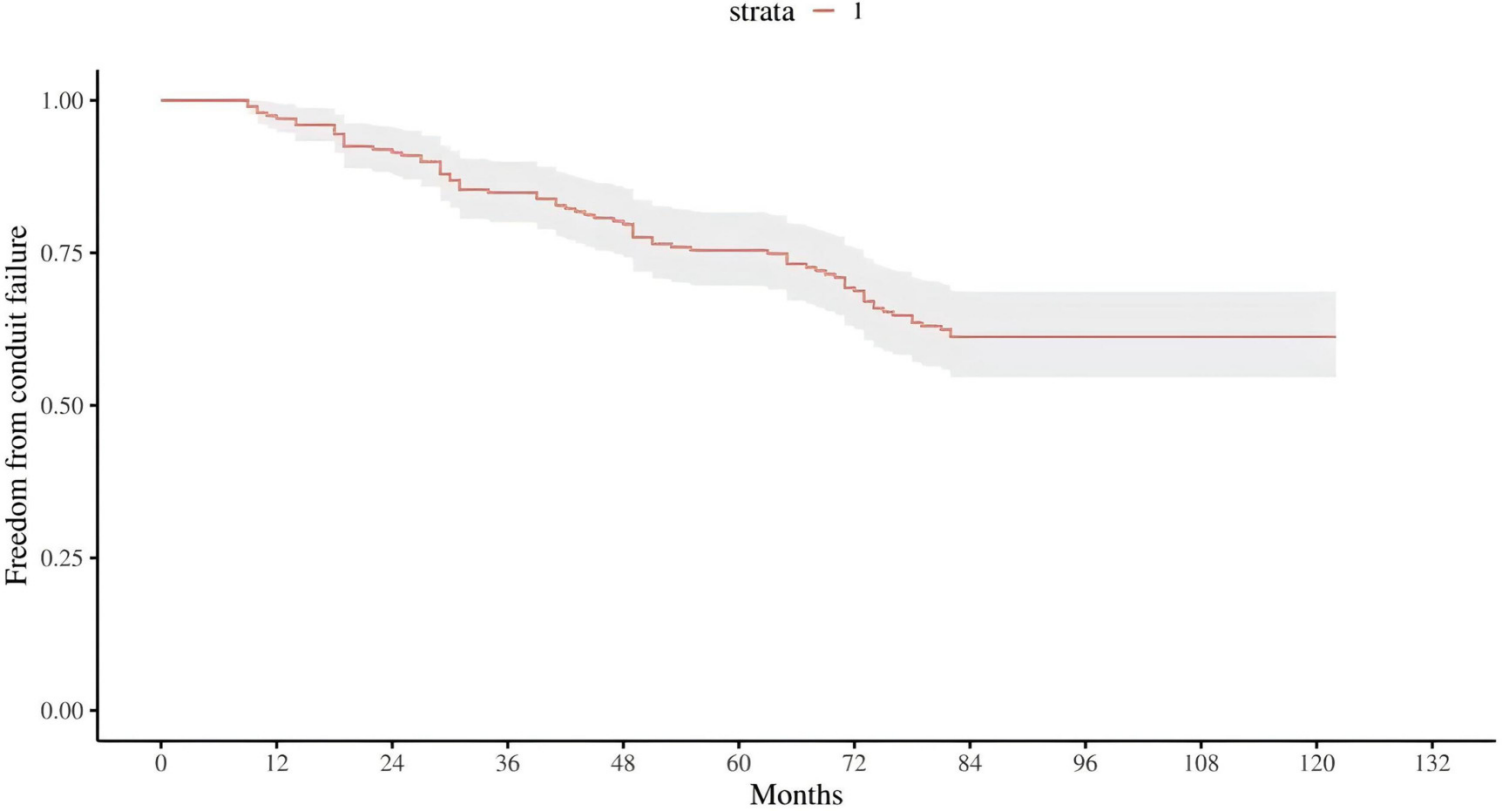
Freedom from BJVC failure, kaplan–meier curve for the entire cohort.

**Figure 2:**
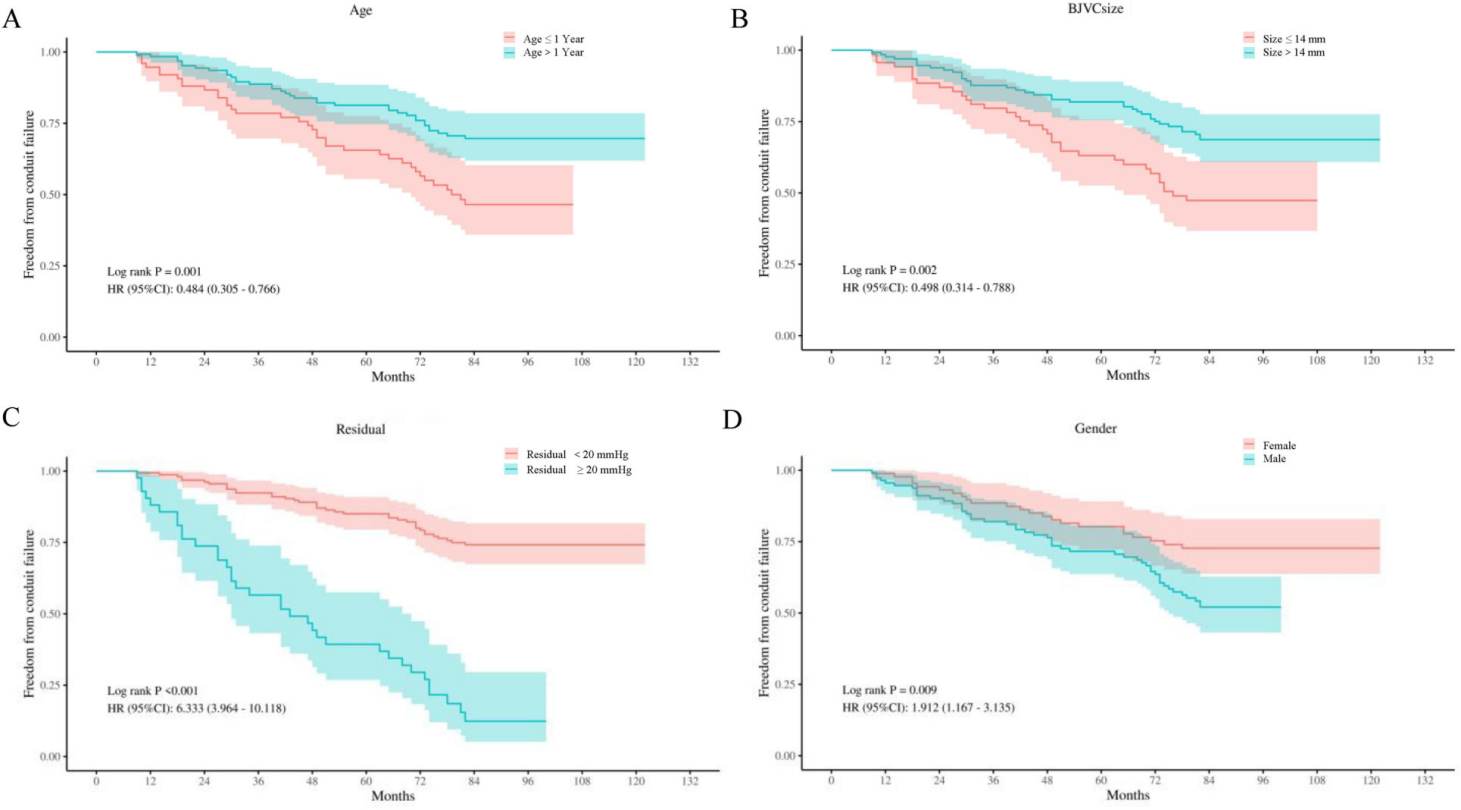
Kaplan–Meier curves of freedom from conduit failure stratified by **(A)** age group (≤1 year vs. >1 year), **(B)** BJVC size (≤14 mm vs. >14 mm), **(C)** postoperitive residual gradient (<20 vs. ≥20 mmHg), and **(D)** sex (female vs. male). Log-rank *p*-values shown.

During follow-up, 29 conduit failures (37.2%) occurred: isolated stenosis in 16 (20.5%), combined stenosis with regurgitation in 6 (7.6%), isolated regurgitation in 3 (3.8%), and conduit-related infective endocarditis in 4 (5.1%). Diagnosis-stratified counts are summarized in Supplementary Figure S2. Kaplan–Meier curves showed high early durability with progressive late attrition: estimated freedom from failure was 97.4%, 87.2%, 75.6%, and 62.8% at 1, 3, 5, and 7 years, respectively (Figure 1). Subgroup curves separated in patterns concordant with the multivariable model: patients with a postoperative residual gradient ≥20 mmHg had substantially lower freedom from failure than those with <20 mmHg; smaller conduits (≤14 mm) and age ≤1 year at implantation also exhibited poorer durability relative to their reference groups, whereas male sex showed an intermediate but visibly inferior trajectory compared with females (Figure 2).

### Univariable predictors of BJVC failure

On univariable Cox analysis, a postoperative residual gradient ≥20 mmHg was strongly associated with higher failure hazard (HR 6.33, 95% CI 3.96–10.12; *P* < 0.001). Male sex conferred higher hazard than female (HR 1.91, 95% CI 1.17–3.13; *P* = 0.010). Longer operative times were associated with higher hazard: CPB time per minute (HR 1.02, 95% CI 1.01–1.03; *P* < 0.001) and cross-clamp time per minute (HR 1.04, 95% CI 1.02–1.05; *P* < 0.001). Infants (age ≤1 year) had a higher risk of conduit failure than older children, whereas age >1 year was associated with a lower hazard compared with ≤1 year (HR 0.48, 95% CI 0.31–0.77; *P* = 0.002). Similarly, smaller conduits (≤14 mm) were at higher risk, and a conduit diameter >14 mm was associated with a lower hazard compared with ≤14 mm (HR 0.36, 95% CI 0.16–0.79; *P* = 0.011). Pre-operative diagnosis categories were not significantly associated with failure when compared with pulmonary atresia (reference). Laboratory indices and other peri-/post-operative course variables were not significantly associated. Univariable results are shown in Table 2.

**Table 2:** Univariable cox analysis of predictors for BJVC failure.

Variable block	Level/predictor	HR (95% CI)	*P* value
Conduit diameter (mm)	>14	0.36 (0.18–0.71)	0.003
	≤14	1.00	
Age (y)	>1	0.48 (0.31–0.77)	0.002
	≤1	1.00	
Sex	Male	1.91 (1.17–3.13)	0.010
	Female	1.00	
Postoperative residual gradient (mmHg)	≥20	6.33 (3.96–10.12)	<0.001
	<20	1.00	
CPB time		1.02 (1.01–1.03)	<0.001
Aortic cross-clamp time		1.04 (1.02–1.05)	<0.001
Pre-operative diagnosis	TGA/VSD/PS	1.83 (0.67–5.00)	0.239
	DORV	1.39 (0.32–6.06)	0.661
	Truncus arteriosus	4.71 (0.73–30.34)	0.103
	TOF	0.95 (0.13–7.15)	0.959
	PA	1	
RBC		0.96 (0.79–1.15)	0.644
WBC		0.97 (0.88–1.06)	0.488
CRP		1.32 (0.79–2.20)	0.285
Hb		1.00 (0.99–1.01)	0.734
LVDD		0.97 (0.93–1.02)	0.202
LVEF		0.98 (0.87–1.11)	0.783
RVDD		0.97 (0.93–1.01)	0.143
Oxygen saturation		0.99 (0.97–1.01)	0.440
Intraoperative blood loss		1.32 (0.56–3.10)	0.520
Length of stay		0.89 (0.21–3.77)	0.878
ICU stay		1.13 (0.87–1.47)	0.365
Mechanical ventilation time		1.44 (0.87–2.38)	0.154

RBC, red blood cell count; WBC, white blood cell count; CRP, C-reactive protein; Hb, hemoglobin; LVDD, left-ventricular diastolic diameter; LVEF, left-ventricular ejection fraction; RVDD, right-ventricular diastolic diameter.

### Multivariable predictors of BJVC failure

**Figure 3:**
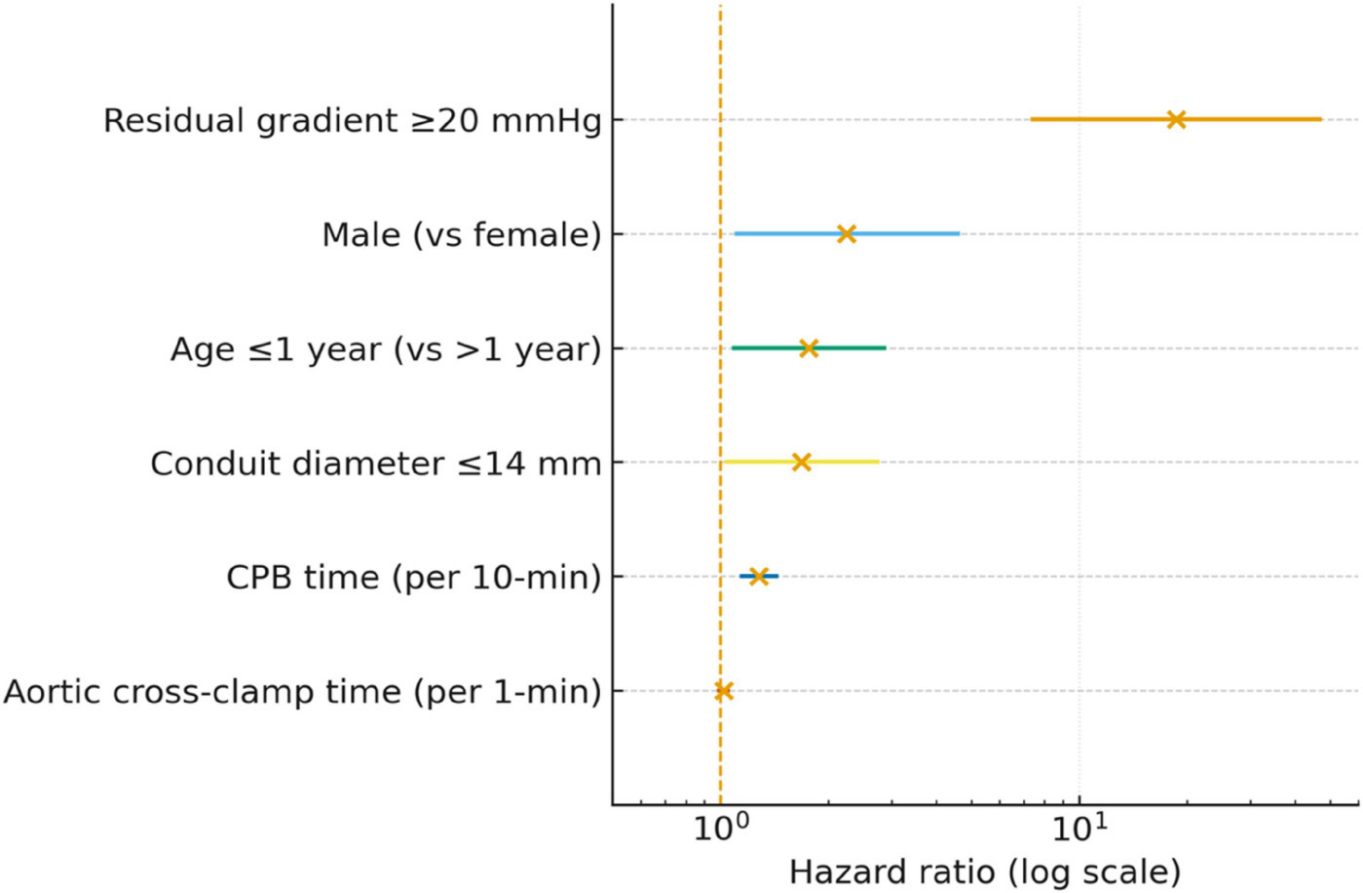
Multivariable forest plot for BJVC conduit outcomes.

In the multivariable Cox model, five predictors were independently associated with BJVC failure: residual gradient ≥20 mmHg (HR 18.67, 95% CI 7.43–46.94; *P* < 0.001), male sex (HR 2.25, 95% CI 1.11–4.58; *P* = 0.025), age ≤1 year (HR 1.76, 95% CI 1.09–2.86; *P* = 0.022), conduit diameter ≤14 mm (HR 1.68, 95% CI 1.04–2.74; *P* = 0.035), and longer CPB time. For interpretability, CPB was scaled per 10 min (HR 1.28, 95% CI 1.15–1.43; *P* < 0.001). Aortic cross-clamp time was not independently associated (HR 1.02, 95% CI 0.94–1.04; *P* = 0.44). Age ≤1 year and conduit diameter ≤14 mm remained independent risk factors for BJVC failure, consistent with the univariable findings that older age and larger conduits are associated with lower hazards. These results support the clinical observation that infants and smaller conduits represent a more vulnerable subgroup. Multivariable estimates are presented in Table 3, with a forest plot in Figure 3.

**Table 3:** Multivariable cox analysis of predictors for BJVC failure.

Characteristics	HR (95% CI)	*P* value
Residual gradient ≥20 mmHg	18.67 (7.43–46.94)	<0.001
Male (vs. female)	2.25 (1.11–4.58)	0.025
Age ≤1 year (vs. >1 year)	1.76 (1.09–2.86)	0.022
Conduit diameter ≤14 mm	1.68 (1.04–2.74)	0.035
CPB time (per 10 min increase)	1.28 (1.15–1.43)	<0.001
Aortic cross-clamp time	1.02 (0.94–1.04)	0.44

### Variable selection with LASSO

**Figure 4:**
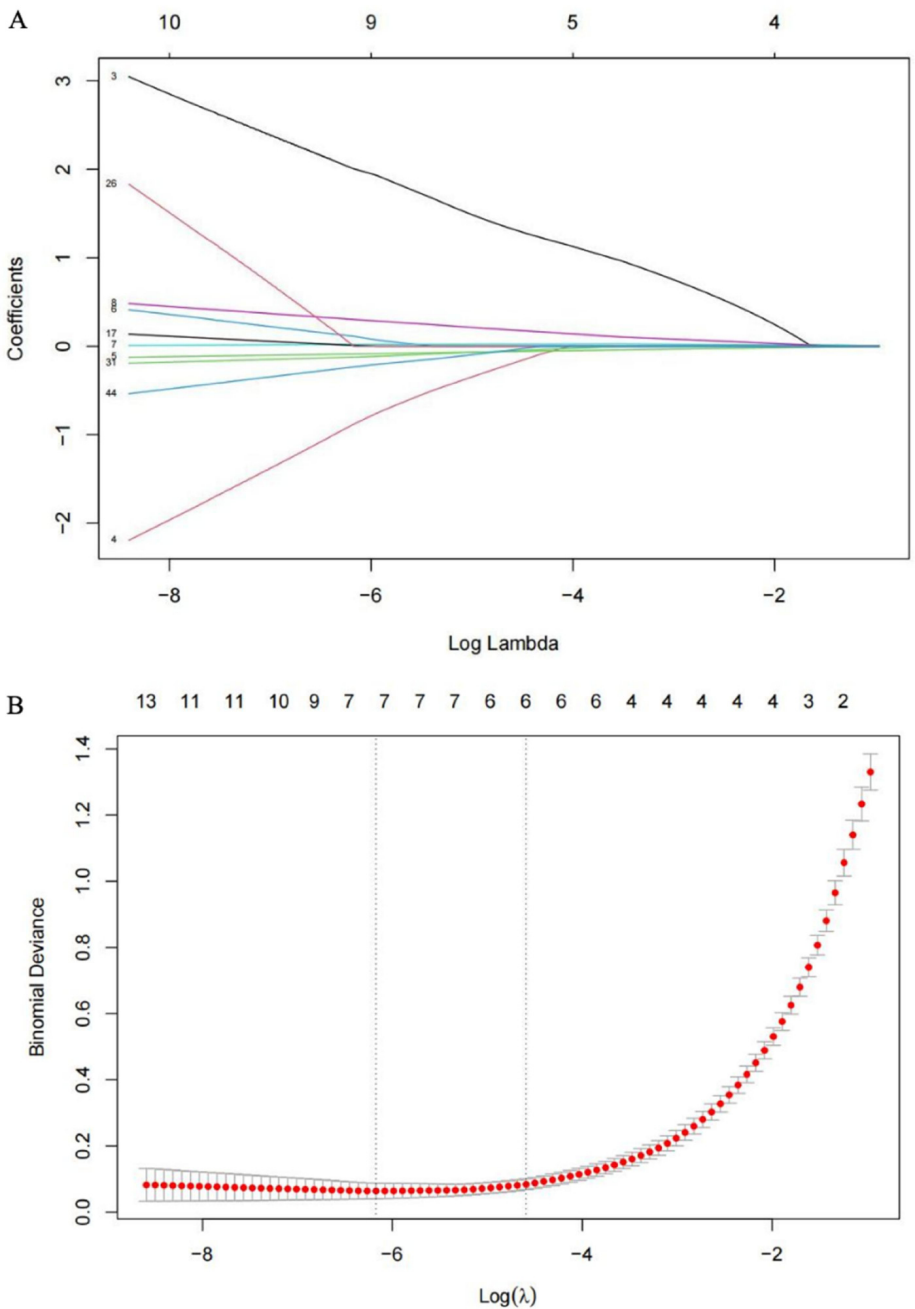
Proﬁle charts for the LASSO model. **(A)** LASSO coefficient paths for candidate predictors; **(B)** Cross-validation curve for LASSO.

Using LASSO with 5-fold cross-validation, the following predictors had non-zero coefficients at the optimal penalty and were advanced for modeling: age, sex, CPB time, conduit diameter, pre-discharge residual gradient, aortic cross-clamp time, and RV end-diastolic dimension (RVDD) (Figures 4A,B). Directions of LASSO-shrunk coefficients were clinically plausible (higher risk with greater residual gradient and longer CPB; lower risk with larger conduit diameter).

### Nomogram construction

**Figure 5:**
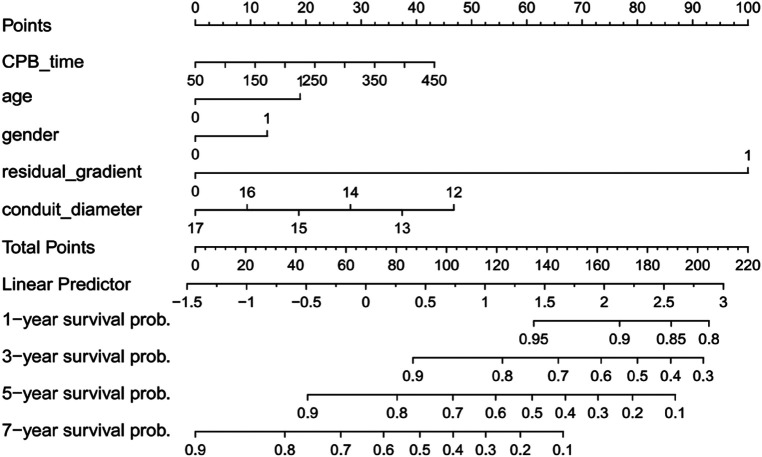
Nomogram for predicting freedom from conduit failure at 1, 3, 5, and 7 years. Points are assigned for each predictor and summed to yield time-specific survival probabilities.

A nomogram was constructed from predictors retained in the multivariable model—residual gradient (≥20 vs. <20 mmHg), sex (male vs. female), age (≤1 vs. >1 year), conduit diameter (≤14 vs. >14 mm), and CPB time (per 10 min increase)—to estimate individualized 1-, 3-, 5-, and 7-year freedom from failure probabilities (Figure 5). Each variable contributes points on the “Points” axis; the sum maps to the linear predictor and then to time-specific survival probabilities. Higher total points indicate lower freedom from failure.

### Performance of the nomogram

**Figure 6:**
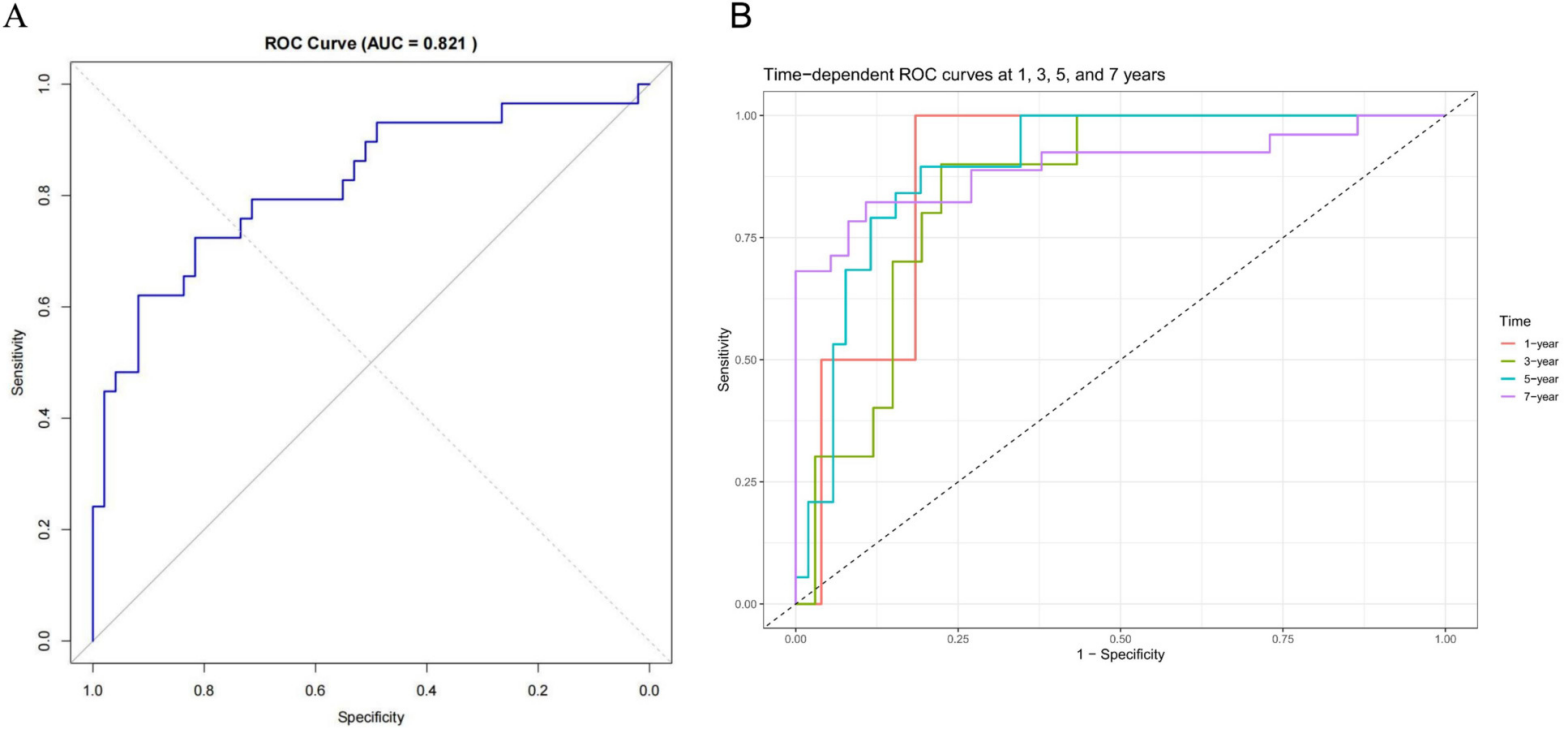
Discriminative performance of the BJVC prediction nomogram. **(A)** Conventional ROC curve for the overall prediction. **(B)** Time-dependent ROC analysis showing AUCs at 1, 3, 5, and 7 years.

**Figure 7:**
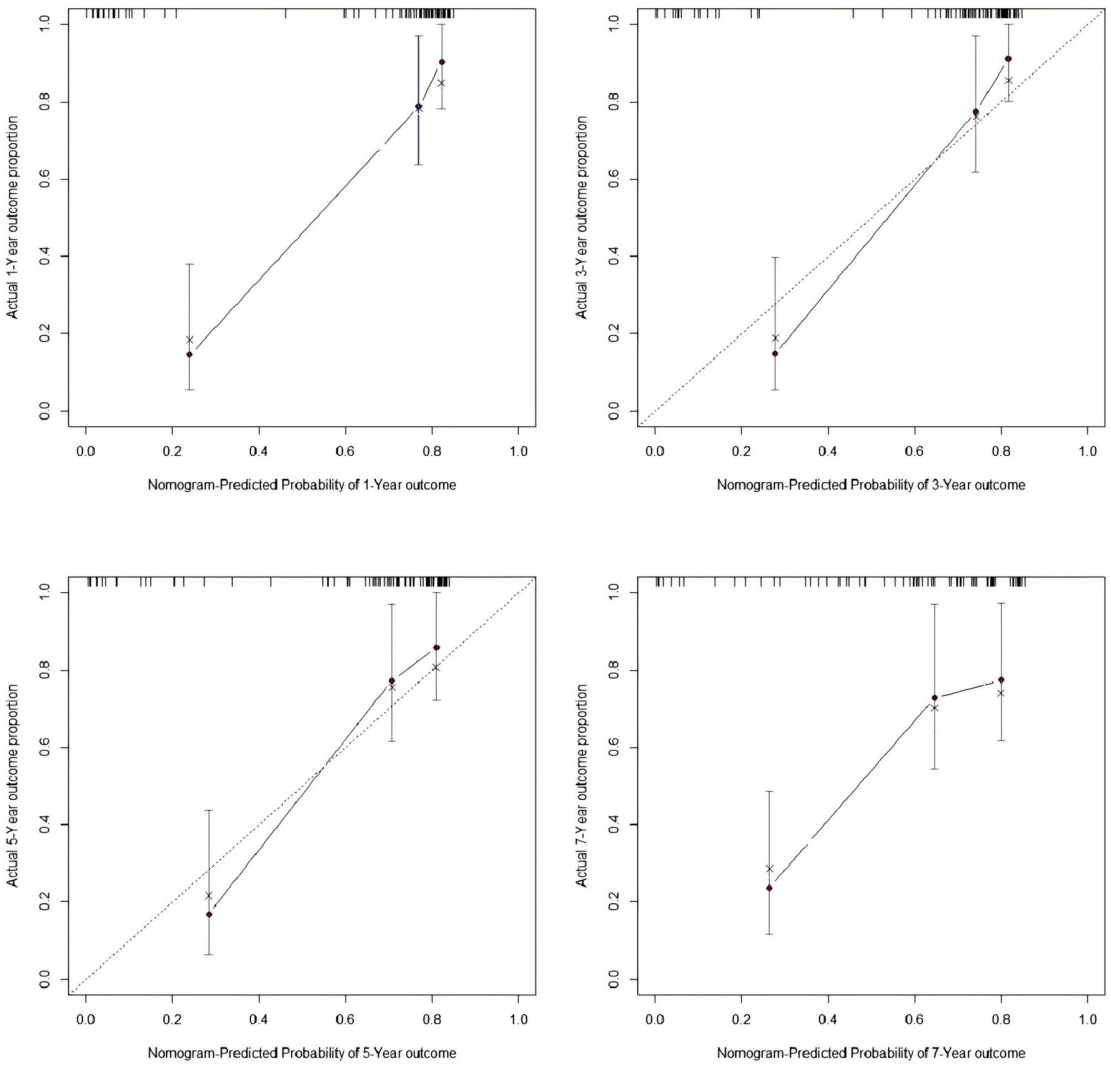
Calibration plots at 1, 3, 5, and 7 years. Observed vs. predicted probabilities with the 45° reference; 1- and 3-year curves closely match the ideal line, with modest drift at 5 and 7 years.

The nomogram showed good overall discrimination, with an apparent ROC AUC of 0.821 at the end of follow-up (Figure 6A). In addition, time-dependent ROC analysis demonstrated that the AUCs at 1-, 3-, 5-, and 7-year follow-up were 0.888, 0.850, 0.900 and 0.897, respectively, indicating that the discriminative performance of the model remained high and stable over time (Figure 6B). Calibration was satisfactory at all prespecified horizons: the 1-year and 3-year curves closely tracked the 45° reference line, indicating minimal systematic error, while the 5-year and 7-year curves displayed only modest departures that remained within clinically acceptable bounds (Figure 7). Taken together, these findings indicate that the model provides reliable short- to mid-term risk estimates of freedom from conduit failure and can support postoperative risk stratification and follow-up planning.

## Discussion

In this single-center pediatric cohort undergoing primary BJVC implantation, overall durability was acceptable but declined with time. Freedom from conduit failure was 97.4%, 87.2%, 75.6%, and 62.8% at 1, 3, 5, and 7 years, respectively. A multivariable model identified postoperative residual RVOT gradient, conduit size, age at implantation, sex, and CPB time as the key correlates of outcome, enabling construction of a clinically usable nomogram with good discrimination and satisfactory calibration. These data outline the time course of BJVC degeneration and highlight actionable peri-operative targets.

Infective endocarditis (IE) occurred in 4/78 patients (5.1%), which is comparable to rates reported in prior BJVC series (19). Beckerman et al. observed an approximately 10% cumulative incidence of IE at 7.5 years, with excellent outcomes after surgical replacement (20). Large-scale registry data further indicate a higher IE risk with BJVCs compared with other RVOT conduits (21), whereas the risk appears similar to that seen with Melody valves (10). Importantly, Hosam et al. found no association between IE risk and the mode of BJVC implantation (percutaneous vs. surgical), suggesting that the vulnerability may relate to the xenograft itself rather than the delivery route (22, 23). Proposed mechanisms include delayed endothelialization and biofilm formation on thrombogenic conduit surfaces, as well as local turbulence at suture lines that predisposes to endothelial injury and bacterial adhesion (24). Collectively, these data support vigilant long-term surveillance and a low threshold for timely surgical replacement once IE is confirmed.

Severe regurgitation was uncommon in our series and showed little progression over time, although prior reports vary (25). Both our data and others link higher right-ventricular pressure (RV-LV pressure ratio >0.6 or RV pressure ≈ 100 mmHg) to conduit dilation and secondary regurgitation, underscoring caution when residual RV pressure is expected to remain high (26). By contrast, conduit stenosis constituted the predominant adverse endpoint and tended to progress with time, arising from anastomotic narrowing, intimal hyperplasia, calcification, and thrombosis (27). Practical mitigation strategies include meticulous anastomotic technique and, as suggested by Prior et al., thorough pre-implant flushing to remove residual glutaraldehyde and dampen inflammatory responses (28).

The dominant effect of residual gradient ≥20 mmHg in our model is clinically actionable. Persistent obstruction plausibly sustains adverse shear and promotes intimal hyperplasia and leaflet restriction, accelerating degeneration (29). Accordingly, geometry-conscious implantation (commissural alignment, suture-line orientation, avoidance of distal kinking), early hemodynamic optimization, and a low threshold for catheter-based relief if gradients persist are reasonable targets. Our finding that small conduits (≤14 mm) fare worse is consistent with pediatric series showing that younger age/low weight/smaller size predict earlier dysfunction via velocity and wall-stress penalties during growth (30, 31). While aggressive “upsizing” must respect annular and branch-PA constraints, contemporary data caution that oversizing is not a universal panacea and may itself carry early adverse signals—supporting a largest-feasible-but-physiologic sizing strategy individualized to anatomy (4, 32, 33). Parallel biomaterials-oriented reviews highlight avenues that could modulate surface–microbe interactions and host remodeling, helping reconcile the material-linked IE susceptibility with hemodynamic factors (4).

Male sex and age ≤1 year likely combine biological susceptibility with exposure. Infants accumulate mismatch between a fixed-caliber graft and an enlarging vascular bed; differences in immune maturation and calcium-phosphate handling may prime earlier fibro-calcific change. Large single/multicenter experiences confirm that pediatric RV-PA conduit failure reflects a composite of patient, material, and size factors, with sex or age remaining independent signals in adjusted models (34). Longer bypass time is not directly “treatable,” but it indexes operative complexity and systemic inflammation that can injure endothelium and prime pro-fibrotic pathways; acknowledging it as a risk cue justifies intensified early surveillance (31). Intriguingly, recent multi-conduit cohorts suggest that non-homograft conduits (including BJVC) fail earlier than homografts, and that anticoagulation may attenuate gradient progression in selected settings—hypotheses that merit prospective testing rather than protocol adoption at this stage (35).

Translationally, the nomogram operationalizes these insights at the point of care. High-risk profiles (infant, male, ≤14 mm conduit, residual gradient near/above 20 mmHg, longer CPB) warrant compressed echocardiographic intervals during the first 24 months, predefined thresholds for catheter intervention, and earlier discussion of redo strategies before fixed RV remodeling. Conversely, low-risk profiles can follow standard surveillance, minimizing unnecessary imaging burden without compromising safety. This tiered approach is aligned with contemporary RVOT management guidance and facilitates shared decision-making with families (36). Recognizing that any model may slightly over-predict in the extreme risk tail, periodic recalibration or dynamic updating with longitudinal gradients/RV metrics should improve absolute risk estimates over time; similar nomogram frameworks have proven implementable in pediatric cardiac populations (37).

Several limitations warrant consideration. First, the single-center, retrospective design and modest sample size may limit external generalizability and reduce power to detect more subtle effects (e.g., anatomic variants or technical nuances). Although internal performance was acceptable, optimism bias cannot be excluded; external, multicenter validation and prospective model updating are required before broader clinical use. Second, the number of failure events (*n* = 29) relative to the five predictors in the final model yielded an EPV of approximately 5.8, below the conventional recommendation of at least 10–15 events per variable. This low EPV may increase the risk of overfitting and unstable coefficient estimates. Although we used penalized variable selection and bootstrap internal validation to partially mitigate this issue, the present model should be regarded as hypothesis-generating and requires confirmation in larger, independent cohorts. Third, event adjudication relied on standardized echocardiographic thresholds and clinical review; misclassification remains possible despite protocolized follow-up. To address these limitations—particularly the lack of external validation—we are preparing a multicenter study that will enroll consecutive children undergoing primary BJVC implantation at several tertiary pediatric cardiac centers. Patients will be followed prospectively, and the current risk model will be applied to evaluate discrimination, calibration, and clinical utility across institutions. Depending on its performance, the model may be updated or recalibrated before any routine clinical implementation.

Looking ahead, three avenues merit evaluation. First, dynamic risk modeling that incorporates time-updated RVOT gradients and right-ventricular functional indices may refine individual risk trajectories and mitigate edge-zone over-/under-prediction. Second, biomechanics-informed implantation—using conduit-sizing strategies that account for projected somatic growth and flow modeling—could help minimize early residual gradients in high-risk strata. Third, structured surveillance pathways that link risk bands to predefined imaging intervals and interventional thresholds may harmonize follow-up and enable earlier, lower-morbidity reinterventions. In summary, our study delineates a reproducible risk architecture in pediatric BJVC recipients and translates it into a pragmatic toolset. With external validation and periodic recalibration, this approach could align surveillance intensity and intervention timing with individualized risk across childhood.

## Conclusions

In this single-center pediatric cohort undergoing primary BJVC implantation, durability declined over time and failure was driven mainly by stenosis. Five factors independently predicted failure—postoperative residual RVOT gradient ≥20 mmHg, smaller conduit diameter (≤14 mm), age ≤1 year, male sex, and longer CPB time—and were integrated into a nomogram that showed good discrimination and acceptable calibration. These findings highlight residual gradient (modifiable) as a priority target for peri-operative optimization and support use of the nomogram for individualized risk stratification and follow-up planning. Given the retrospective, single-center design, external validation of the model is warranted before wider application.

## Data Availability

The raw data supporting the conclusions of this article will be made available by the authors, without undue reservation.

## Supplementary material

Supplementary figure S1 **(A)** Distribution of missing data values; **(B)** data imputation after missing values were filled in.

Supplementary Figure S2 Conduit failures distribution.
