# Supplementary material for: Risk factors and a nomogram for bovine jugular vein conduit failure after right ventricular outflow tract reconstruction: a 10-year single-center cohort
Source: Front Pediatr. 2026 Feb 6;14:1733522. doi: 10.3389/fped.2026.1733522 (PMC12920508; doi:10.3389/fped.2026.1733522)
Supplement: Supplementary figure S1 — (A) Distribution of missing data values; (B) data imputation after missing values were filled in. [file Presentation1.pdf]

Supplementary Materials

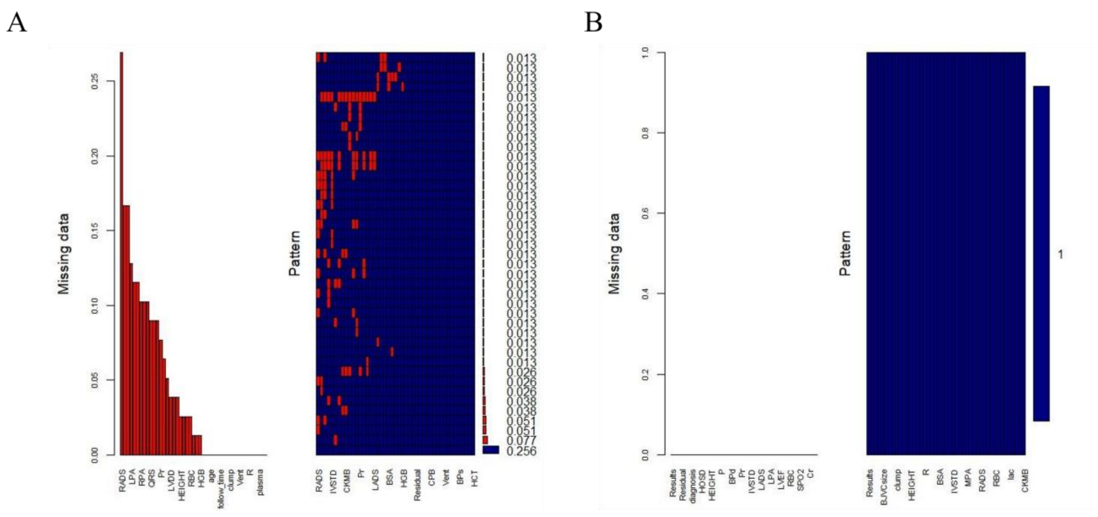

Supplementary figure S1: A. Distribution of missing data values; B. data imputation after missing values were filled in.

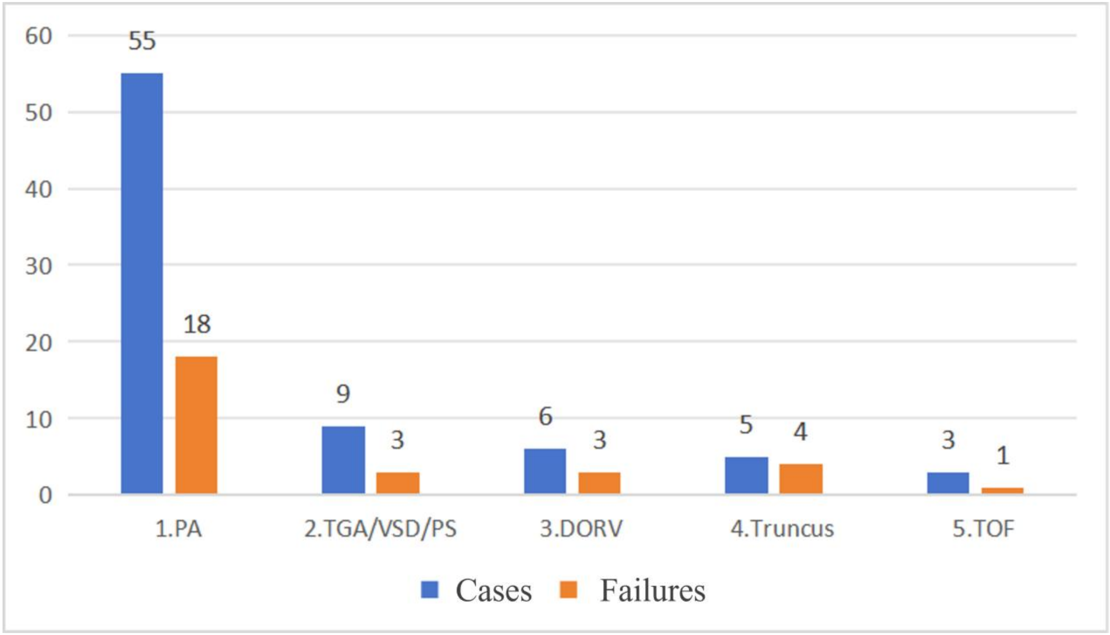

Supplementary Figure S2: Conduit failures distribution.
